# Tracing the Path Toward Self-Regulated Revision: An Interplay of Instructor Feedback, Peer Feedback, and Revision Goals

**DOI:** 10.3389/fpsyg.2020.612088

**Published:** 2021-02-05

**Authors:** Wentao Li, Fuhui Zhang

**Affiliations:** School of Foreign Languages, Northeast Normal University, Changchun, China

**Keywords:** self-regulated revision, instructor feedback, peer feedback, revision goals, EFL writing

## Abstract

Building upon Zimmerman’s socio-cognitive view of self-regulation, we explored EFL (English as a Foreign Language) students’ revision and the likely contribution to revision from three salient self-regulating sources: peer feedback, instructor feedback, and revision goals. Data was obtained from 70 Chinese EFL students in a writing class through a 300-word online writing assignment involving online instructor and peer feedback, free-response revision goals, and a required revision. We closely coded students’ revision and then used the same coding scheme to analyze the relative levels of association of revision changes with peer comments, instructor comments and revision goals. We found that: (a) the majority of revision changes have been triggered by three mediating sources, with revision goals as the most significant contributing source. Additionally, most revision changes come from a combination of two or three sources, with the overlap of peer feedback and revision goals accounting for the biggest overlapping contribution for both high and low-level revisions; (b) as for the relationship among the three sources, no significant difference was found between revision goals’ overlap rate with peer feedback and their overlap rate with instructor feedback. Instructor feedback and peer feedback did not overlap very much. Findings suggest that students could revise beyond instructor and peer feedback in their revision efforts guided by their own reflective goals, and peer feedback could function as a more productive and multiple-reader source of revision in comparison with instructor feedback. This study also provided evidence for students’ self-regulated learning of writing through the use of self-regulating resources and charted a route for how writing could be improved.

## Introduction

A number of studies have found that students could achieve more when involved in self-regulated learning of writing processes like goal setting and feedback (Zimmerman, 2000; Teng and Zhang, 2016; Panadero, 2017; Zhang, 2018). Goals can shape writing performance by marshaling effort to address specific writing issues, increasing persistence, and motivating students to utilize other strategies and social resources (Graham et al., 1992; Zimmerman and Kitsantas, 1999; Cumming, 2006; Bown, 2009; Topping, 2009; Hyland, 2016). Feedback quite often comes from instructors and peer students. Instructor feedback, as a powerful and useful resource in the revision process, has led to writing improvement over drafts (Hyland, 2003; Hyland and Hyland, 2006a; Ferris, 2010). Peer feedback, as another important resource, can invoke reflection for self-assessment and improve EFL writing performance (Min, 2006; Topping, 2009; Boud and Molloy, 2013; Lee, 2017; Zhang, 2018; Li and Zhang, 2019). However, most of the previous studies centered on strategies’ effects independent of each other or one strategy’s effect over another, and the combined effects of the salient self-regulated strategies on writing and revision are seldom explored, even though they quite often appear together in EFL writing context and work together toward self-regulated learning (Liu and Carless, 2006; Min, 2006; Yang et al., 2006; Min, 2016; Hu and Gao, 2018).

Self-regulated learning has several models which commonly share three salient elements: goal, feedback, and actions (Zimmerman, 2002; Panadero, 2017; Schunk and Greene, 2018). From a socio-cognitive perspective of self-regulated learning of writing, three salient elements can interact with each other: personal regulation strategies like writing goals, environmental regulation strategies like peer and instructor feedback as well as behavioral regulation strategies like revising (Zimmerman and Risemberg, 1997; Zimmerman, 2013; Ziegler, 2014; Zhang et al., 2017).

From a pragmatic point of view, EFL writing learning needs all available learning sources in harness with each other to improve students’ writing performance. Instructor feedback and peer feedback as crucial sources of information scaffold learner’s learning experience, feed into learner uptake, but they often fail to generate expected results, as learners’ feedback literacy or engagement with feedback vary (Han and Hyland, 2015; Han and Xu, 2019; Yu et al., 2019). On the one hand, learners could integrate more instructor feedback than peer feedback into revision, for they viewed instructor feedback as more authoritative or valid (Caulk, 1994; Yang et al., 2006). On the other hand, learners could adopt more peer feedback because it was better understood than instructor feedback (Zhao, 2010). Contrasting these different findings, we found an important relevant factor was not present. What were the students’ own goals for revision? The reflective learning goals probably mediate the effects of external feedback on revising behavior. To bridge the gap in between, students’ revision goals need to be elicited, which could be an essential learner factor in contributing to revising actions.

In the current study, self-regulated revision refers to revision based upon students’ adaptive use of personal, environmental, or behavioral regulation strategy (Zhang et al., 2017). Students could use environmental strategies like instructor and peer feedback and personal strategies such as writing goals to regulate revising behaviors, thereby approaching their self-regulated learning of writing (Zimmerman, 1995, 2013; Zhang et al., 2017, 2019; Zhang, 2018). As Zimmerman noted, the self-regulated writing is a dynamic and intricate process with constant interaction of three self-regulated strategies. Whereas the whole working mechanism might not be uncovered in one study, salient ingredients like writing goals, instructor feedback, and peer feedback can be elicited to delve into their combined effects on revision and the possible interactive relationships among the regulating sources.

## Literature Review

### A Socio-Cognitive View of Writing Self-Regulation: The Theoretical Framework

The concept of self-regulation emerged from Bandura’s seminal social cognitive theory (Zimmerman and Schunk, 2003). Writing development entails self-regulation, because writing is often “self-planned, self-initiated, self-sustained” and self-constructed (Zimmerman and Risemberg, 1997, p. 76). Nested in Bandura’s socio-cognitive theory, writing self-regulation is decomposed into three forms of self-regulated learning: personal, environmental, and behavioral, and then integrated into an ongoing interaction of a triadic cycle (Zimmerman and Risemberg, 1997). Later, while more self-regulation models were generated with focus on motivation or metacognition (Winne and Hadwin, 1998; Pintrich, 1999; Pintrich et al., 2000; Panadero, 2017), they share several elements with various names in explaining the whole cycle of self-regulated learning: goals, feedback, and actions. As Zimmerman said in an interview (Panadero, 2017), “cognitive processes bi-directionally cause and are caused by behavior and environment”. For example, goal setting at the very beginning of the writing might guide the drafting of students, while later feedback probably feed into writers’ knowledge and skills of writing and stimulates another round of goal setting, namely revision goals, which, in turn, guides the second round of writing behavior, to be more specific, revising.

These three processes interact reciprocally, as writing and revising are cyclical and recursive rather than neat or linear. Environmental regulation such as instructor feedback and peer feedback could be filtered into students’ writing uptake, enriching their writing schemata. Naturally, students reflect through writing, reviewing, and receiving review from instructors and peers, and reformulate their personal goals which guide students to monitor their revising performance, and revising is critical to bring writing into alignment with writers’ goals (Sommers, 1980).

Little is known about how the three main sources interact to promote self-regulation: instructor feedback, peer feedback, revision goals. In a typical EFL writing classroom, all three sources could be present to maximize the learning benefits, though peer review and goal setting are so far not practiced in every learning context. Pairwise overlap between the sources may be more predictive of revising behavior than issues in only one source in a learning context. If the overlap occurs too rarely to account for much revision, students might change their writing goals too drastically in their revising behaviors. The current study is an observational study of instructor feedback, peer feedback, revision goals and revision to help build a holistic account of self-regulated learning theory about the role of personal goal setting and environmental feedback from instructors and peers.

**FIGURE 1:**
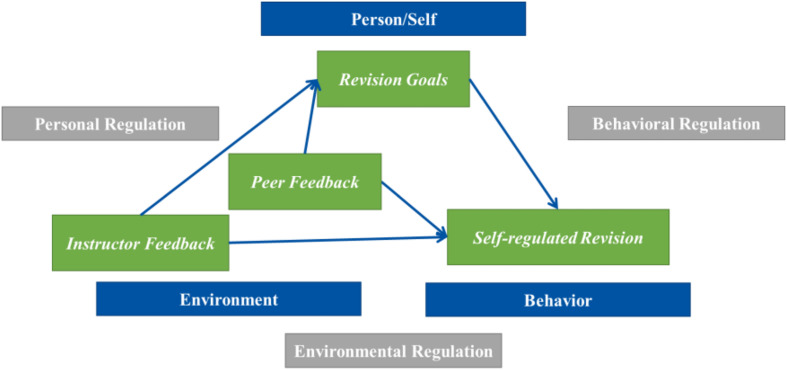
Theoretical framework of writing self-regulated learning activities through the triadic causational sources.

Three essential components fall into the current study’s scope: revision goal setting as personal self-regulation, feedback from peers and instructor as environmental self-regulation, and writing performance and revision as behavioral self-regulation (see Figure 1).

### Instructor Feedback and Self-Regulated Writing

Feedback is one of the most potent determiners in learning and achievement and is an essential catalyst for all self-regulated activities (Butler and Winne, 1995; Hattie and Timperley, 2007; Carless et al., 2011; Hawe and Dixon, 2017; Xu and Carless, 2017; Gan, 2020; Wisniewski et al., 2020). It is conceptualized as information given by a social agent (e.g., self, instructor, peer, book, etc.) regarding aspects of one’s task performance (Kluger and Denisi, 1996; Hattie and Timperley, 2007; Duijnhouwer et al., 2010; Carless and Boud, 2018; Carless, 2019). As Zimmerman (1989, 2001, 2008) noted, although self-regulated learning of writing is viewed as especially important during personally directed forms of learning such as self-selected reading, it is also deemed very important in social forms of learning writing, like receiving accessible feedback from instructors and peers.

Among the external sources, the written/oral mode of instructor feedback is considered the dominant kind of feedback in EFL writing classroom. EFL writing instructors usually spend massive amount of time writing feedback on students’ texts, thus providing useful mediation for students to improve their writing particularly in the context of tertiary and secondary level education (Ferris, 2014; Lee, 2017; Yu, 2019). Studies on instructor feedback center on three themes including written corrective feedback (WCF), written commentary, and oral feedback.

Early research on instructor written feedback consistently indicated that EFL writing instructors viewed writing as a product only and focused predominantly on language errors/mistakes in students’ writing (Cumming, 1985; Zamel, 1985; Ferris, 2003). Since the introduction of the process approach in EFL writing pedagogy, more studies shifted the focus from low-level dimension like language form to high-level issues such as content, organization, and logic (e.g., Saito, 1994; Ferris, 1995). For example, Ferris (1995, 1997), and her team’s research (Ferris et al., 1997) demonstrated that only 15% of instructor feedback addressed grammar and mechanics, while 85% focused on content and rhetorical development. Biber et al. (2011) meta-analysis of instructor feedback in EFL writing suggested that a balanced focus on content and form was more effective than focusing on language form alone. Multiple studies suggest the benefits of a more selective/focused approach to WCF (Sheen et al., 2009). Equally important, and perhaps even more, is instructors’ concrete and reflective written commentary (Goldstein, 2004; Hyland and Hyland, 2006b). In order to increase students’ uptake, building relationships with students by having face-to-face oral communication to negotiate meaning and giving individualized feedback produces aggregational effects (Ferris, 2014; Lee, 2017; Carless, 2019).

However, in many EFL writing classrooms, especially in a context where instructor feedback resources are very limited in contrast to the large number of students in need of writing and rewriting practice, instructor feedback might have focused on the student’s shared writing needs or main issues in student essays, instead of elaborate comments. To compensate, students may turn to other agents such as classmates/peers for diversified feedback and suggestions to facilitate self-regulated writing (Zhang et al., 2020).

### Peer Feedback and Self-Regulated Writing

Peer feedback, sometimes also called peer assessment, peer review or peer evaluation, is a social strategy that has been widely used in disciplinary courses including natural science, the humanities and the social sciences (e.g., Topping, 2005, 2009; Cho et al., 2006; Yu and Lee, 2015; Alitto et al., 2016; Lee, 2017; Chang et al., 2020; Wu and Schunn, 2020; Zhang et al., 2020). In the meantime, Learning sciences shape peer feedback as a more and more core concept from “Assessment of Learning” (AoL) to “Assessment for Learning” (AfL) (Carless, 2005; Lee and Coniam, 2013; Butler, 2018), and finally to “Assessment as Learning” (AaL) (Boud and Molloy, 2013; Lee, 2017). Especially in EFL writing studies, scaffolded peer feedback has been largely practiced and its effect was mainly found in students’ progress in cognitive, social, and linguistic development (e.g., Liu and Hansen, 2002; Liu and Carless, 2006; Guardado and Shi, 2007; Hsu and Hsu, 2016; Yu and Hu, 2017; Zhang, 2018; Lee and Evans, 2019; Lin, 2019; Zhang et al., 2019; Zhou et al., 2020).

Most important, feedback from peers can facilitate students’ writing progress by prompting EFL students to reflect on their learning by giving useful comments, for example gaining in-depth understanding about task demands, and gathering more strategies and skills to enhance their own writing (Lundstrom and Baker, 2009; DiDonato, 2013; Min, 2016; Hsu et al., 2018; Li et al., 2020). Students can also learn to treat evaluations as opportunities for deepening understanding and sharing/refining self-regulated learning strategies (DiDonato, 2013; Zhang, 2018).

Despite the extensive use of the peer assessment activities in teaching EFL writing, questions that concern the accuracy and helpfulness of peer feedback are continually posed. In particular, a number of investigations have demonstrated that peer feedback can trigger meaningful revisions as instructor review does if peers are guided by clear rubrics and held accountable for the quality of the feedback they received (Berg, 1999; Topping, 2005; Cho et al., 2006; Gielen et al., 2010; Panadero et al., 2013; Schunn et al., 2016). However, some studies pointed out the problems in the peer assessment process. For instance, untrained or lack of experienced peer feedback tended to overly concern about the problems on low-level language, including grammar, vocabulary, and punctuation (local aspects) or ignored the problem of high-level content and organization (global aspects) in peers’ writing (Min, 2005, 2006; Altstaedter, 2018).

With the development of educational technology, EFL students are more engaged with increasingly complex multimodal texts for different purposes and audiences (Dzekoe, 2017; Qu, 2017). Multimodal composing has been widely practiced to help EFL students participate in online peer feedback activities and gain positive experiences (see Cho and MacArthur, 2010; Manchón, 2017; Gao et al., 2019; Unsworth and Mills, 2020). Typically, some online technologies like online peer review systems (Peerceptiv, Eli, Peergrade, etc.) and social media learning systems (QQ, Wechat, etc.) provide multimodal learning spaces that can facilitate students’ self-regulated learning of writing processes (Zhang et al., 2019). Those technologies largely help reduce the logistics of paper sharing, integrate peer assessment into diverse assignments, empower students to assess as teaching-learning partners, and thus accelerate the pace of self-regulated writing.

### Goal Setting and Self-Regulated Writing

The process of writing has long been recognized as a characteristically goal-oriented activity (Graham and Harris, 1994). Goal setting involves specifying the intended outcomes of writing efforts (Zimmerman, 1997; Schunk, 1990). Students use goals to regulate themselves through the extended mental effort required to coordinate and direct their thinking while they compose. Goal itself is rather a psychological term. Paris et al. (2001) asserted that goals are self-constructed theories of self-competence based on both internal and external sources of information, involving desires and actions in respect to personal estimations of possible selves, satisfaction about performance, standards for judging and modifying these, and feedback from others. In other words, goals are goal setter’s self-proposed outcome based upon information from external and internal sources. Goals can be either in line with instructional goals or not, since they are basically constructed by students themselves.

Goal has been viewed by educational psychologists such as Schunk (1990) and Zimmerman (2001) as a focal component of self-regulated learning across all disciplines (Middleton and Midgley, 1997; Pajares et al., 2000). In particular, students can use goals to monitor and improve writing performance (Flower and Hayes, 1981; Bogolin et al., 2003; Cho, 2017; Yilmaz Soylu et al., 2017). Students might have unique personal goals for one piece of writing and somewhat constant goals for developing writing abilities over time (Flower and Hayes, 1981; Zhang et al., 2017). Goals for writing and writing improvement differ among different students or different cultural norms and expectations in various types of texts and situations (Heath, 1983; Connor, 1996).

Prior research has found that goal setting as an effective self-regulated strategy can improve students’ writing performance across school levels (Graham et al., 1992; Zimmerman and Kitsantas, 1999; Crews and Aragon, 2007; Ferretti et al., 2009). Silver (2013) argued that the use of specific writing goals in the self-edit step not only increased writing quality but also writing quantity in experiments with children of average achievement. Bogolin et al. (2003) demonstrated that 5th-grade students improved their writing performance through setting writing goals based upon the writing rubrics such as support, and organization. In terms of undergraduate novice students, MacArthur and Philippakos (2013) found that by using goal strategy instruction over two semesters, substantial gains in writing achievement and motivation were found, especially in the second academic cycle, and students who have goals focused on learning to write rather than on grades were more likely to be successful in later classes. The above studies suggest the benefits of the use of specific/explicit goals for revision across proficiency levels.

Although the importance of writing goals may be self-evident, many unanswered questions lie ahead. First, previous learning goal settings were mostly given directly by the writing instructors or generated under the guidance of the instructors. The writing instructors positioned themselves in the roles of the student’s savior and interventionist. They assume that students lack specific learning goals (e.g., Schunk and Swartz, 1993a, b; Ferreti et al., 2000; Bogolin et al., 2003; Silver, 2013). Nevertheless, students, especially university students, are likely to have their own writing goals in a given assignment (though contextualized by the assigned task), and the focus of the student-generated goals may differ from instructor generated goals, even confined to a specific writing goal category. Investigating self-generated goals’ effects on writing, Zhang et al. (2017) discovered that students revised their drafts guided by their initial writing goals. However, they might change their writing goals over drafts, especially when mediated by peer feedback intervention in between. After all, students’ general writing goal for the first draft is to get the writing task done. Little is known about student’s goals for the revised draft and how they might exert effects on their writing.

### The Relationship Among the Self-Regulating Variables

Despite many variables in students’ self-regulated learning of writing processes (i.e., goal setting, instructor feedback, peer feedback, self-reflection, contingent reinforcement, etc.), the relationship among some salient self-regulating variables remains an interesting issue to be explored. An older meta-analysis conducted by Schunk (2003) investigated 39 studies and found that a combination of goal setting with other self-regulated variables such as feedback, and self-assessment can facilitate students’ self-efficacy for self-regulated reading and writing. In the follow-up research studies, they discovered that goal setting, together with contingent reward or peer-triggered feedback, had positive effects on writing performance. For example, in Hansen and Wills (2014) case study, the interplay of contingent reward and goal setting intervention increased the number of correctly spelled words (from 27.3 to 37.4) in one elementary school student’s writing. Alitto et al. (2016) found that 114 elementary school students receiving the goal setting and peer-mediated feedback intervention performed significantly higher on production-dependent writing indices (total words written, words spelled correctly, and correct word sequences) than control groups (ES = 0.12–0.28).

A more recent study conducted by Zhang et al. (2017) found that when students being introduced to a combination of self-regulated strategies like goal setting, peer feedback, and self-reflections from peer feedback, students enhanced their writing performance (paper rating for the second draft improved by a mean of 0.21 out of 7, ES = 0.54) and made revisions in both high (content) and low-level (language) dimensions especially when writing goals overlap with peer comments. No instructor feedback was involved in the self-regulated process of disciplinary writing in that study. However, instructor feedback is obviously very common in EFL writing class.

Much self-regulated writing research focuses on L1 English students, while few studies take EFL students into account; they are a vast population still struggling with writing and revision (e.g., Li and Zhang, 2019; Zhang et al., 2020). What are the features of revision goals for EFL students, and what are the effects of revision goals as they interplay with instructor feedback and peer feedback concertedly acting on revision? These concerns need to be further studied.

In a nutshell, issues concerning the interaction among the variables (i.e., revision goals; instructor feedback; peer feedback; self-regulated revision, etc.) in the process of EFL writing are still open to debate. The current study, rooted in Zimmerman’s socio-cognitive view of writing self-regulation, explored how students regulated their writing via feedback from the instructor and peers and their own revision goals.

## Research Questions of the Present Study

Specifically, we asked foundational questions on their self-regulated revising behaviors and how the socio-cognitive strategies including revision goals, instructor feedback, and peer feedback contribute to revision. The questions are as follows.

RQ1: Has the writing grade improved over drafts? How was student revision distributed across rating dimensions?
RQ2: Have the revisions been addressed by self-regulated sources including peer feedback, instructor feedback, and revision goals? Are there any revisions triggered by multiple self-regulated sources?
RQ3: To what extent are instructor feedback, peer feedback, and revision goals related?

## Methods

### Research Context

The current study was conducted within a 70 freshmen course entitled ‘English Grammar and Writing’ in a B.A. program in English language at a key university in Northeastern China with around 30,000 students. The focus of the course is to develop English major students’ fundamental English writing skills such as narration and description, and language skills such as sentence construction and styling. The course was given by a teacher researcher with ten years of English writing teaching experience.

The teaching and learning of writing basically followed Zimmerman (2000) socio-cognitive model of writing self-regulation: personal regulation, environmental regulation, and behavioral regulation. The model entails the inherent cycle of social learning from others and then self-monitoring through cognitive and behavioral efforts. As the model points out, learning writing started from observing others’ writing in the context of the study. Prior to the writing phase and the peer assessment activities the instructor gave instructions on genre writing through close reading and in-class discussion of how the model essay followed the genre structure. For the present essay centering on a kind of emotion, a model essay is “The Yellow Ribbon” in which the hero’s anxiety is a main clue of the story. Then, students would set the thematic goal and drafted a narrative essay centered on a certain predominant emotion of themselves.

**FIGURE 2:**
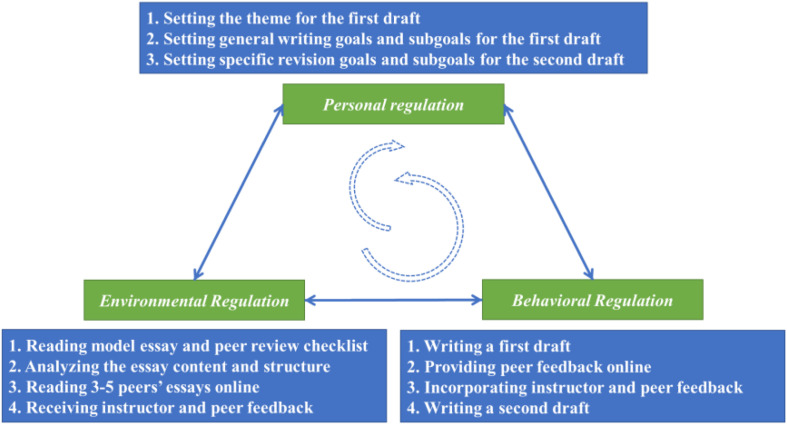
Cycle of writing self-regulated learning activities through three causational sources.

Across writing assignments, students were taught, trained on, and checked on four essential writing skills: unity, support, coherence, and wording/sentence skills. When they were first trained about the four skills, they were given a sample writing and a peer review checklist adapted from Langan (2011: 225) (see Appendix B for details). The instructor let students pick out problems first, and then help students use the peer review checklist to identify more relevant problems. Later, students were required to review the first draft of 3-5 peers’ writing across assignments and provide peer feedback online. When students’ review came to an end, the instructor gave each student feedback too. Receiving both instructor and peer feedback, students decided to incorporate useful feedback they have received and setting specific revision goals, and writing a second draft. Students practiced writing and revision skills across writing assignments and gradually approached writing self-regulation (see Figure 2 for details). The regulating process is highly dynamic with interactive regulating factors. One interesting regulation episode is how students draw insights from social sources and set their own goals to improve their writing performance. To what extent students incorporate teacher and student feedback into revision goals and revision later is the central issue in the current study.

The writing assignment involved in the study was done as a second one. The writing task adapted from Langan (2011: 223) required students to write an essay of approximately 300 words to narrate an experience in which a certain emotion was predominant. The emotion might be disappointment, embarrassment, fear, happiness, love, nervousness, sadness, regret, etc. The chosen experience was to be limited in time (see Appendix A for details).

Students were required to use an online platform *Tencent QQ* to submit their writing assignment. *Tencent QQ* (more popularly known as QQ) is a conversation-based instant messaging social media platform. It can be used to converse with others through private message interface and group message interface. Although the QQ platform is not a platform established specifically for writing, it is a convenient and frequently used communication tool for college-level EFL students (Zhao, 2015; Zhang et al., 2019). Students as writers can upload their writing files and respond to them synchronically and reciprocally in a group message interface, via oral or written messaging. Students as reviewers were required to evaluate at least three consecutive files according to the random order of submission. Instructor feedback was also provided in the same group message interface. After receiving both instructor and peer reviews, students set revision goals, revised their essays, and turned in a second draft along with their revision goals on top of the paper, again to the QQ platform.

### Participants

Seventy participants (89% female, around 18 years old) enrolled in the course were all native speaker of Mandarin Chinese, and they have learned English for at least twelve years, and got English test scores ranging between 110 and 150 in the entrance examination to Chinese university with a full score of 150 (upper-intermediate level across the country). Their previous writing learning experiences were confined to around 150 words of notices or short letters. Students’ writing performance were rated by two research assistants. Students who completed all four steps of activity including first draft submission, instructor and peer assessment, revision goal elicitation, and second draft submission fall into 53 among the previous 70. For those who were dropped out, most of them forgot to write their revision goals, which were not accounted for in their final grade.

### Measures

#### Revision Work

**FIGURE 3:**
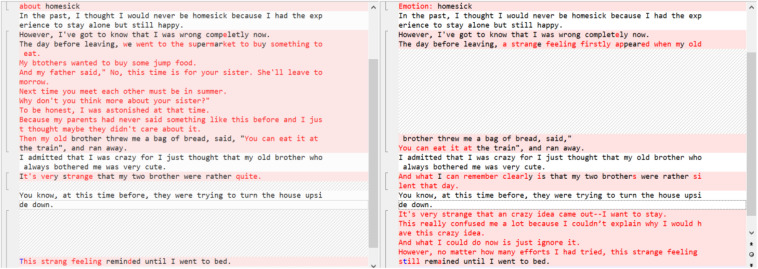
Illustrative example of comparison between two drafts.

In order to track students’ revision work systematically, we used *Beyondcompare 4* (a multi-platform utility that combines directory compare and file compare functions in one package) to compare the changes between the first draft and second draft as well as label each revision according to a coding scheme with definitive description and revision examples (see Figure 3). The highlighted revisions include both high-level content changes (such as clarity and support, coherence, and cohesion) and low-level language changes (such as grammar, word choice, and spelling). To label them, we coded them by hand by two research assistants.

The categories of revision were generated by means of iterative comparison to refine the coding across two random subsample sets (50 revisions for each) by one research assistant. Categories were inductively developed that elicited the focus of students’ revision. The tentatively coded categories were then used to try on the remaining revision samples, with improvement to the coding until the resulting coding scheme produced a working definition for each category and several illustrative examples for revision.

These preliminary revision definitions and examples were provided to a second research assistant as a reference for the first round of inter-coder reliability trial. The coding scheme was then revised by adding to and integrating some existing categories. This revised revision coding scheme is presented in Table 1. It was assessed for inter-rater reliability, producing acceptable Kappas all above 0.65 on all, and strong Kappas near or above 0.8 for all (see Table 1 for details). 0.65 is quite acceptable in the current study, because the writing task has no definite topic to be addressed, and such writing evaluation is acceptable usually at 0.4 (Shrout and Fleiss, 1979).

**TABLE 1:** Coding scheme for revision work, peer feedback, instructor feedback, and revision goals, with coding reliability Kappas in parentheses.

Revision category	High/low	Revision description	Revision examples	Peer feedback examples	Instructor feedback examples	Revision goals examples
Clarity and support	High	Using specific, typical, and adequate evidence to support the central evidence, illustrating personal, real life, and relatable details	(K = 0.94) Add specific, relative details: My mother kept quiet and gave me a deep hug to encourage me. That night I stayed up late all night. I felt so nervous and fearful.	(K = 0.91) I think it would be better if you can add more details to describe the emotion.	(K = 0.96) More conversations about your physical and emotional interactions would make it more interesting to read!	(K = 0.88) Add more emotional description about my central topic, support more details.
Coherence and cohesion	High	Presenting strong connection, good flow, good transitions, and clear order	(K = 1) Change the order of sentences: As we approached, we noticed that there were a group of people in the circle. Getting closer, we found two young men singing in the crowd. My friend…	(K = 1) The context connection needs to be reprocessed.	(K = 1) Maybe for the last but one paragraph, the first two sentences reverse their order, the story will be more coherent!	(K = 0.90) Reverse the order of some sentences.
Unity	High	Covering a clear central idea, crossing off irrelevant content	(K = 0.65) Delete irrelevant content: Humans are emotional. They are influenced by many emotions such as…	(K = 1) I think the last two sentence in the third paragraph can be deleted because it is about the overcoming and has nothing to do with fear.	(K = 0.90) The first paragraph is not so relevant to the story. I think that part can be deleted.	(K = 1) Some unrelated things could be omitted to make the focal points stand out.
Task response	High	Addressing all parts of the writing task and better achieving the writing requirements	(K = 1) Change the topic: Freedom → Missing.	(K = 1) I think the topic “freedom” is more like a kind of state than an emotion so it seems to be a bit off topic.	(K = 1) Focusing on one emotion is ok. Two might be more distracting.	(K = 1) Revise the task response.
Wording	Low	Word choice, adding/deleting/replacing appropriate word(s) for clarity	(K = 0.97) Add a word for clarity: He suggested us to go to Xiangya hospital for a further and more professional examinations.	(K = 1) As for the second paragraph, I think this “but” is not very appropriate. You can say like this: “Freedom don’t mean…, but (means)…	(K = 0.85) Pay attention to words like rain cats and dogs, mute/silent, similar to with. Check dictionaries.	(K = 0.90) Use more advanced words to improve the article.
Grammar	Low	Using standard written English with good mechanics(e.g. the proper use of tenses, the passive voice, and modal auxiliaries)	(K = 0.96) Correct the tense error: At first my friend and I went into the first room, there were (are) a table, a box, many toys, and a cupboard.	(K = 1) Your article has few errors about grammar such as “I am can’t still understanding”.	(K = 0.90) Pay close attention to the grammar errors. “leave your mac lonely” need to change into “leave … alone”, I guess.	(K = 1) I’ll pay attention to my grammar problems.
Sentence skills	Low	Arranging sentences grammatically, syntactically, and structurally	(K = 1) Segmentation: I fell into a deep panic, Went into another room, Hid behind the wardrobe, And called my father to tell him to go back quickly.	(K = 0.92) I think it may be better to simplify those long sentences because readers are likely to skip sentences if they are too long.	(K = 1) Pay attention to some sentence structures, like we could say words failed me in expressing my love and sweetness.	(K = 1) Pay attention to the correct usage and collection of the sentence patterns.
Spelling	Low	Forming English word correctly from individual letters	(K = 1) Correct the spelling error: This strange (strang) feeling still remained until…	(K = 0.85) Typing too fast might lead to spelling mistakes.	(K = 1) Pay attention to the spelling problem, like “September”.	(K = 1) Try to reduce the spelling errors.
Title/subtitle	High/low	Making specific changes to the title/subtitle	(K = 1) Add a title: *Calm* Add a subtitle: *If Miss Will Have The Voice*	(K = 1) Maybe indicating the emotion on the title is necessary.	(K = N/A) N/A	(K = 1) Indicate the emotion on the title.

#### Feedback From Peer and Instructor Assessment

The feedback from peers and the instructor served as relevant self-regulated sources toward revision. Each student was to review at least two essays and provide written feedback on their peers’ first draft on the QQ platform by directly messaging their peers. Similarly, the instructor gave written feedback in the same way. Altogether, 53 pieces of instructor feedback (one for each student) and 143 pieces of peer feedback (one to five for each student) were produced for these 53 students. The length of peer comments is usually at least as long as instructor feedback and most of them longer. The overlap between revision and the instructor and peer feedback were coded using the same categories (see Table 2 for examples). The inter-rater reliability on 100 random peer comments produced strong Kappas near or above 0.8 for all. As for coding instructor feedback, the coding scheme was assessed for inter-rater reliability on 53 instructor comments, producing strong Kappas near or above 0.8 for all except one category (title/subtitle) which was deem not applicable (see Table 1 for details).

**TABLE 2:** Illustrative examples of the overlap between peer and instructor feedback.

Feedback category	High/low	Peer feedback	Instructor feedback
Clarity and support	High	I think if you add more details such us your conversation with your dad and environmental description, it will be better to outstand your emotion.	More conversations about your physical and emotional interactions would make it more interesting to read!
Coherence and cohesion	High	The sentence connection needs to be reprocessed, some of them did not make sense, focusing on the coherence.	Maybe for the last but one paragraph, the first two sentences reverse their order, the story will be more coherent!
Unity	High	Your first paragraph has nothing to do with the emotion.	The first paragraph is not so relevant to the story. I think that part can be deleted.
Task response	High	I think that you should pay attention to describe your core emotion which is calm instead of using more words on nervous.	Focus on one emotion is ok. Two might be more distracting.
Wording	Low	The only disadvantage in your article is there are some problems on the usage of words, I will suggest you to check an authentic dictionary.	Pay attention to words like rain cats and dogs, mute/silent, similar to with. Check dictionaries.
Grammar	Low	Your article has some grammatical mistakes.	Pay close attention to the grammar errors. “leave your mac lonely” need to change into “leave … alone”, I guess.
Sentence skills	Low	Some sentences did not make sense to me, please do revise (simplify) your sentence pattern and structure.	Pay attention to some sentence structures, like we could say words failed me in expressing my love and sweetness.
Spelling	Low	Typing too fast might lead to spelling mistakes.	Pay attention to the spelling problem, like “September”.

#### Revision Goals

Students were asked to describe their revision goals for the second draft after receiving peer and instructor feedback. Revision goals ranged from as brief as an imperative phrase to as long as a one hundred word one or two paragraphs. The revision goals were coded using the same categories applied to the revisions, and 161 revision goals were found from these 53 students. The content overlap between revision and revision goals was assessed for inter-rater reliability for 100 random examples, producing strong Kappas near or above 0.8 for all (see Table 1 for details).

#### Paper Ratings

One of our research concerns was the improvement of writing performance, so we obtained scores for the two drafts to weigh the difference. The scores were generated from the mean value of two expert reviewers’ ratings (afterward for the research study) on shared rubrics [rating Kappa (1st draft) = 0.91, Kappa (2nd draft) = 0.88]. The rubrics involved different dimensions including centering on a prominent emotion in an experience (unity), elaborating about the experience and the central emotion with supporting details (clarity and support), and connecting the details together logically with natural flow (cohesion and coherence), and attending to low-level aspects of writing (grammar, wording, spelling, and sentence skills). Each dimension accounted for 25% of the overall writing score (see Appendix C for complete reviewing details).

### Procedure

Students were instructed to submit the first draft to the QQ platform, then to provide feedback for at least three peers’ essays, and finally to receive peer and instructor feedback on the QQ platform. Peer feedback was given first, and instructor feedback later. Peer feedback was not confined to English, as the purpose was to cooperate for better writing. However, they all wrote in English, except for few words they could not spell out. When they published peer feedback in the public forum, they usually add @ to address the writer and peer reviewers usually received a conventional thank you message from peer writers, sometimes with simple emoticons. There is no further response to peer comments as far as the writing was concerned.

After receiving both peer and instructor feedback, students were asked to set revision goals for second drafts, and finally to submit the second draft with revision goals written on top of the draft to the QQ platform. Since we wished to learn about students’ self-generated revision goals, no detailed guidance or examples of the revision goals were provided and no word or number limit of goal writing was required. Each phase (submission, online assessment, goal elicitation and revision, and resubmission) took approximately one week.

## Results and Discussion

As noted earlier, in this study, we addressed three research questions, RQ1 exploring student’s revision quality and quantity, RQ2 focusing on contributions of sources of self-regulated revision, and RQ3 further investigating the possible internal interactions between the three sources.

### Has the Writing Grade Improved Over Drafts? How Was Student Revision Distributed Across Rating Dimensions?

In order to measure the student’s revision quantity and quality, we start with basic descriptive statistics regarding revision-triggered writing improvement. A paired sample *t*-test [*t*(52) = 18.3, *p* < 0.000, Cohen’s *d* = 1.51] of paper rating between drafts shows that paper ratings improve by a mean of 0.62 (out of 7). A one-way ANOVA further confirmed a significant change of paper rating between draft [*F*(12, 40) = 12.145, *p* < 0.000, partial η*^2^* = 0.925], suggesting that students’ revision work generated significant improvement between drafts.

From the 53 students, a total of 450 revision changes (252 were high-level revisions, 198 were low-level revisions) were produced between drafts. Each student made an average of 8.5 revisions (noting that some revisions could affect multiple paragraphs). This implies that students made substantial targeted revisions in a 300-word essay.

Among the revision categories, clarity and support (47%), wording (25%), and grammar (16%) were the most common revision categories, while coherence and cohesion (6%), sentence skills (2%), spelling (1%), title/subtitle (1%), task response (<1%), and unity (<1%) were much less frequent. Two cases are coded as “others” because they did not fit these categories (e.g., change the subject of the whole essay: I - Simon). In terms of high-level versus low-level revisions, 56% of the revision changes were high-level revisions, and 44% were low-level revisions. Those revisions improved the essay quality in terms of both content and language. Where those revisions might come from was explored in the next part.

### Have the Revisions Been Addressed by Self-Regulated Sources Including Peer Feedback, Instructor Feedback, and Revision Goals?

Revision changes could be traced from multiple self-regulated sources: peer feedback, instructor feedback, and revision goals. Each revision change was compared by hand against the three sources for content overlap (see Table 3 for examples of each type of overlap). 22% of revision changes could not be associated with any of the three sources.

**TABLE 3:** Illustrative examples of revisions and their likely sources.

Ex.#	Revisions	Revision category	High/low	Peer feedback/Instructor feedback/Revision goals content
**Peer feedback examples**				
1	Add two paragraphs	Clarity and support	High	Clear and vivid are the best points of the whole you’re writing. And I think it would be even better if you add more on the detailed description of your emotion and make the words for storytelling shorter.
2	Delete two sentences for clarification	Clarity and support	High	A very touching home-sick feeling. All the thoughts in your mind on the train make sense to the topic. The only problem I think is the first paragraph is too long and you can polish the first paragraph a little on your impatient with your parents.
3	Add conjunctions	Coherence and cohesion	High	With your great writing skills, I really feel your feelings. But in my opinion, you can add some connectives to your writing to make it more coherent.
4	Fix tense errors	Grammar	Low	Your piece of writing is wonderful, I think there are many details to show your emotion. Just pay attention to some tense errors and it will be perfect.
5	Combine short sentences into a long sentence	Sentence skills	Low	The feelings of your article are sincere, and the changing process of mental activities was also fully demonstrated. I think what can be improved is that more short sentences can be integrated into long sentences in order to make the article more compact.
**Instructor feedback examples**				
1	Delete three irrelevant sentences	Unity	High	The first paragraph is not so relevant to the story. I think that part can be deleted.
2	Add three sentences for elaboration	Clarity and support	High	Memorable childhood with many sweet memories of fun! Great! That book was a secret so far. Maybe elaboration could start from that book about childhood.
3	Change the order of two sentences	Coherence and cohesion	High	Very interesting story to read! I am touched by the understanding and pure friendship you two shared. Maybe for the last but one paragraph, the first two sentences reverse their order, the story will be more coherent!
4	Change wording	Wording	Low	Very touching father-daughter love story! I enjoy reading it! Pay attention to words like rain cats and dogs, mute/silent, similar to with. Check dictionaries.
5	Fix tense errors	Grammar	Low	Pay close attention to the tense/grammar errors like regretful, tiring…
**Revision goals examples**				
1	Choose a new topic	Task response	High	Revise the task response.
2	Add few sentences and words for elaboration	Clarity and support	High	Adding more details when I received the message can make my writing more vivid.
3	Add two conjunctions	Coherence and cohesion	High	More conjunctions, more flow.
4	Fix tense errors	Grammar	Low	Correct few grammar mistakes.
5	Add a title	Title/subtitle	Low	Indicate the emotion on the title.

**FIGURE 4:**
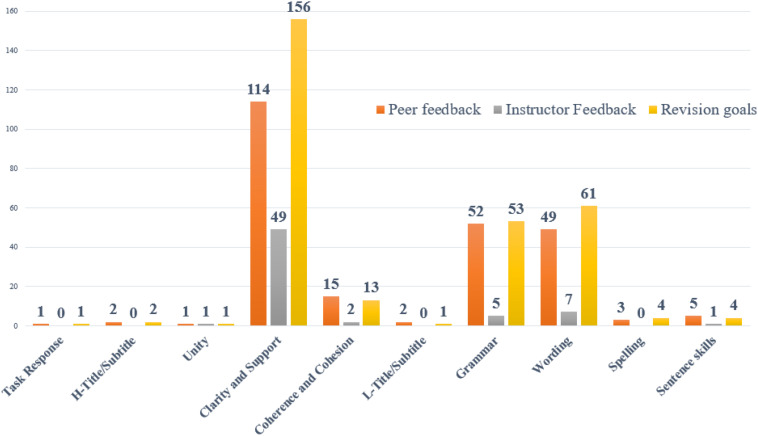
Quantitative distribution of revisions and their likely sources.

Among the rest 348 (78%) retrievable revision changes (199 high and 149 low-level revisions), 70% of revision changes were associated with peer feedback, 19% were associated with instructor feedback, 85% were associated with revision goals (see Figure 4 for the distribution of revisions).

### Are There Any Revisions Triggered by Multiple Self-Regulated Sources?

**FIGURE 5:**
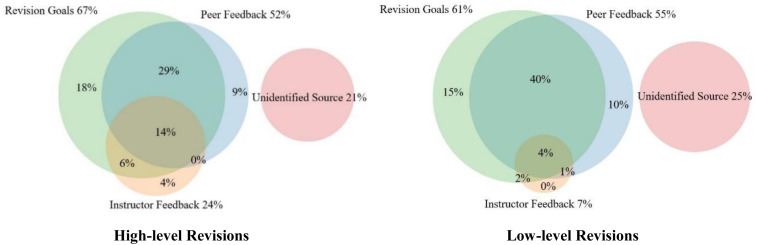
Relative distribution of likely sources of high and low-level revisions.

Among the 348 (78%) retrievable revision changes, 30% of revision changes could be traced from only one source; 48% came from a combination of peer feedback, instructor feedback, and revision goals, and 22% of revision changes came from no source. In terms of high and low-level revisions, Figure 5 presents a Venn diagram of the relative levels of association of peer feedback, instructor feedback, and revision goals with high and low-level revisions.

79% of high-level revisions and 75% of low-level revisions could be traced from at least one of the three sources. High-level revision changes were associated with revision goals most (67%), with peer feedback second (52%), and with instructor feedback third (24%), as Figure 5 shows. It is worth noting that revision goals and peer feedback were more contributive to revision than instructor feedback in both high and low-level revision.

A salient feature demonstrated by the Venn diagram for both high and low-level revisions is that a large percentage (35% for high-level and 42% for low-level) of the revisions were associated with two sources. Additionally, 14% of high-level revisions were associated with all three sources. By contrast, only 4% of low-level changes were associated with all three sources. For both high and low-level revisions, a revision was most likely to be associated with the combination of peer feedback and revision goals. For low-level revisions, 40% of revisions come from peer feedback and revision goals. It is also worth noting that high-level revisions were not likely to be associated with the combination of instructor feedback and peer feedback, and this pattern can also be found in low-level revisions. In summary, both high and low-level revisions appear to be dependent on having multiple sources of input.

The robustness of the revision source distribution patterns shown in Figure 5 can be tested by further inferential statistics. We calculate the relative frequency of association of revisions with each source combination (i.e., a cell in the Venn) for each student (e.g., for a student, what proportion of their high-level revision changes were overlapping with peer feedback but not with either instructor feedback or revision goals?). Then, we use a paired sample t-test to contrast the relative cell sizes by source inside a type of revision (e.g., peer feedback versus instructor feedback for high-level revision changes; see Table 4). The relative magnitudes of effects are shown using Cohen’s *d*. Clearly, the relative high-level contribution of revision goals was biggest, with peer feedback second, and instructor feedback least. In terms of low-level contribution, revision goals and peer feedback made no difference to low-level revision changes.

**TABLE 4:** Relative frequency of sources associated revisions and the pairwise source contrasts.

Revision type	Peer feedback (%)	Instructor feedback (%)	Revision goals (%)	PF versus IF	IF versus RG	PF versus RG
High-level	52	24	67	*t* = 3.4	*t* = 5.0	*t* = 2.7
				*p* < 0.001	*p* < 0.000	*p* < 0.01
				*d* = 0.64	*d* = 0.95	*d* = 0.28
Low-level	55	7	61	*t* = 5.6	*t* = 5.2	n.s.
				*p* < 0.000	*p* < 0.000	
				*d* = 1.00	*d* = 0.95	

The above results only took three separate sources into consideration. Another approach to examine Venn diagram descriptive numbers is to contrast each source’s and the overlap of sources’ contribution to high versus low-level revisions (see Table 5). As a within-subjects t-test on the proportion of each student’s high and low-level revisions, the study found the contributions of peer feedback and revision goals were evenly distributed across high and low-level revisions. Instructors focused mainly on high-level revisions.

**TABLE 5:** Relative frequency of high versus low-level revisions associated with each source and the high versus low contrast.

Source	High-level (%)	Low-level (%)	*t*	*p*	Cohen’s *d*
Not identified	21	25	n.s.		
Peer feedback	52	55	n.s.		
Instructor feedback	24	7	4.0	<0.000	0.78
Revision goals	67	61	n.s.		
PF & IF	0	1	n.s.		
IF & RG	6	2	2.6	<0.011	0.49
PF & RG	29	40	n.s.		
PF & IF & RG	14	4	3.0	<0.004	0.63

### To What Extent Are Instructor Feedback, Peer Feedback, and Revision Goals Related?

Revision goals came from a variety of sources. In this study, we analyzed to what extent revision goals are related to the other two social factors. The 131 actualized revision goals (selected from the collection of the 168 revision goals) were scrutinized to identify the overlap with peer feedback and instructor feedback (see Table 6 for examples of each type of overlap). After that, we use a paired sample t-test to contrast the relative effect sizes by sources inside a specific type of revision goal (e.g., peer feedback versus instructor feedback for high-level revision goals; see Table 7).

**TABLE 6:** Illustrative examples of high and low-level revision goals overlapping with instructor feedback or/and peer feedback.

Ex. #	Revision goals	Revision category	Peer feedback/instructor feedback	Peer feedback/instructor feedback content
**High-level revision goals examples**				
1	The context connection needs to be reprocessed.	Coherence and cohesion	Peer feedback	The emotion is great and detailed. And description is vivid. There are no wrong sentences in the whole article but clauses are not used skillfully. The context connection needs to be reprocessed.
2	Adding more details when I received the message can make my piece of writing more vivid.	Clarity and support	Peer feedback	Clear and vivid are the best points of the whole you’re writing. And I think it would be even better if you add more on the detailed description of your emotion.
3	Add more details/Delete irrelevant details to make story vivid.	Clarity and support	Instructor feedback	The addition did make the build-up more vivid! More details about what small things happened might better build up the grief!
4	Delete the first paragraph.	Unity	Instructor feedback	I enjoy reading your conversation parts, which made the story’s theme very clear and touching. However, the first paragraph is not so relevant to the story. I think that part can be deleted.
5	Add more details about my feelings when I am excited.	Clarity and support	Peer feedback & Instructor feedback	PF: I think the theme is not very prominent. Maybe you can add more details. In general, the words are very vivid. IF: Very good choice of the theme! I like the last sentence best! More details about how you feel when you are excited might work better!
**Low-level revision goals examples**				
1	Avoid some simple wording mistakes.	Wording	Peer feedback	There are some small mistakes (word usage) in this composition.
2	Try to reduce the spelling errors.	Spelling	Peer feedback	From your passage I can feel your feeling. It’s touching and sincere. The cohesion and transition are great. But it has some spelling mistakes.
3	Correct few grammar mistakes.	Grammar	Peer feedback	Your piece of writing is wonderful, I think. There are many details to show your emotion. Just pay attention to some tense errors and it will be perfect.
4	I will pay more attention to the use of verb like frown and the tense.	Grammar	Instructor feedback	The story was well-done. The transition from a favor of you into an embarrassing experience created tension in the story. Pay attention to verb forms like frown.
5	Pay more attention to the grammar.	Grammar	Peer feedback & Instructor feedback	PF: You did a good job. I can feel your regret through your writing. And you added some description about the environment to highlight your emotion. If some sentences and the grammar can be used more properly, it will be better. IF: Send this story to you parents, they might understand your feelings now! Pay close attention to the tense/grammar errors like regretful, tiring…

**TABLE 7:** Relative frequency of peer feedback and instructor feedback associated revision goals, statistical significance, and effect size information for source contrasts.

Revision goal type	Revision goals	Actualized revision goals	Actualized revision goals from PF and IF	Peer feedback (%)	Instructor feedback (%)	PF versus IF
High-level	107	95	80	75	51	*t* = 2.6 *p* < 0.012 *d* = 0.51
Low-level	61	58	51	90	20	*t* = 7.9 *p* < 0.000 *d* = 1.98

We can see the greater overlap with peer feedback was statistically significant in contrast to instructor feedback for both high and low-level revision goals. In other words, the overlapping rate of peer feedback was much higher than instructor feedback with revision goals, and this pattern for setting low-level revision goals was even more striking. Besides that, the adoption rate of all peer feedback (86.7%) into revision goals is slightly higher than that of instructor feedback (81.1%) though the difference is not statistically significant. That implied that peer feedback was given serious attention in comparison with instructor feedback when students picked useful suggestions out from among many. For the remaining 37 revision goals which cannot be traced from instructor and peer feedback, they may come from students’ self-regulation of other external sources or self-reflection.

To summarize, most peer feedback and instructor feedback were integrated into 80% of revision goals; and instructor feedback and peer feedback did not overlap except in those areas addressed by revision goals.

## General Discussion

The current work investigated revision and the interplay of three self-regulating sources of revision: revision goals, instructor feedback, and peer feedback. Most previous literature on the three sources of self-regulated revision has tended to examine each variable in isolation, ignoring the fact that writing and revision is very much a multi-factor driven process of self-regulation (e.g., Page-Voth and Graham, 1999; Hyland and Hyland, 2006a; Liu and Carless, 2006; Min, 2006, 2016; Lundstrom and Baker, 2009; Ferris, 2010, 2014; Silver, 2013; Gao et al., 2019; Zhang et al., 2020).

To explore the cumulative effects of instructor feedback, peer feedback, and revision goals, the study generated a coding framework applied across revision changes and all three sources. In contrast to low percentage of single-source triggered revision changes, 49% for high-level and 47% for low-level revisions were associated with two or three sources. It is especially worth noting that both high and low-level revisions were most likely to be associated with the combination of peer feedback and revision goals, occupying the largest category of association in the Venn diagram shown in Figure 5, especially for low-level revisions with 40% of revisions coming from peer feedback plus revision goals. These findings reveal that the aggregation effects of different self-regulated sources in the student’s self-regulated revision process can be quite notable and be used together in improving writing over drafts.

The improvement can be found in both high-level content and low-level language. Considering the content vs. language dilemma in writing feedback (Manchón, 2011; Qu, 2017; Altstaedter, 2018; Matsuda and Xu, 2019; Polio, 2019), our study suggests that by combining different self-regulated strategies for revision, EFL students can revise both content and language for better. Even if instructors focused more on content issues, students balanced their work on both language and content mainly by uptake of peer feedback and targeted revision goal setting with 58% on content and 42% on language issues. The balance of content and language treatment did not only show in the percentage of peer feedback and revision goal distribution. Indeed, students’ overall writing scores improved by a mean of 0.62 (out of 7), and a paired sample t-test [*t*(52) = 8.95, *p* < 0.000, Cohen’s *d* = 1.41] on low-level dimension rating between drafts showed that the low-level language dimension improved by a mean of 0.64 (out of 7), evidencing the improvement in language is not secondary in comparison with content. An implication is that peer feedback and revision goals could be two very effective sources to trigger self-regulated revision on language issues such as grammar, wording, spelling, and sentence skills.

While echoing earlier studies concerning learning/writing goal setting interventions in writing self-regulation (e.g., Page-Voth and Graham, 1999; Zimmerman and Kitsantas, 1999; Silver, 2013; Zhang et al., 2017), this study elicited students’ revision goal after instructor and peer assessment, instead of taking instructor-directed or purely self-initiated goal setting at the beginning of writing as the sources of revision. Compared with previous goal exploration, EFL students’ cognitive regulation (i.e., personal revision goals) can be traced from more environmental regulation sources (i.e., external feedback from peers), and was likely to influence more revision than one single source, as revision goals proved to be a bigger source of revision than mere instructor or peer feedback. Revision goal elicitation motivates students to weigh feedback from instructors and peers, and thus trigger negotiation with external sources and self-reflection to achieve writing self-regulation.

Although the goal setting process incorporated much of peer feedback and instructor feedback, no causal relationship can be established about the revision goals and their sources. Further research could explore the goal elicitation process using procedures such as interview, think-aloud and stimulated recall to validate whether instructor feedback and peer feedback directly triggered the revision goal, and to what extent goals are generated from instructor feedback, peer feedback, and other sources, like self-re-reading/editing correspondingly.

Another interesting discovery about students’ revision goals is that the overlapping rate with peer feedback (75%) was much higher than that with instructor feedback (51%), and this pattern for setting low-level revision goals was even more striking (90% peer feedback vs. 20% instructor feedback overlapping with low-level revision goals). A probable explanation is that instructor feedback in this research context is very content-oriented (52 out of 65 are classified as high-level comments), whereas peer feedback covers a larger number and a broader range of both content (133 out of 244) and language (111 out of 244).

How strongly did the student’s revision goal interact with other sources when acting on revision behavior? While it was rare to find both high-level (content) and low-level (language) revisions that only stemmed from revision goals, nearly two-thirds of all revisions were associated with the revision goals listed by the students. In addition, revision goals were more commonly associated with revisions when there is another associated source (e.g., 18% from revision goals only vs. 49% from revision goals together with instructor feedback and/or peer feedback for high-level revision changes, 15% from revision goals only vs. 46% from revision goals together with instructor feedback and/or peer feedback for low-level revision changes). This suggests that the revision goals build a motivational context for EFL students to make certain kinds of self-regulated revisions, and that other factors are required to consolidate a revision, supporting the claim made by previous literature with respect to the positive effects of goal setting and its combination with another source, like goal setting plus feedback from instructor/peers (Nicol and Macfarlane-Dick, 2006; Alitto et al., 2016), or its combinations with two additional sources like goal setting plus peer feedback and self-efficacy (Schunk and Swartz, 1993a, b), or goal setting plus peer feedback and self-reflection (Zhang et al., 2017) in the process of self-regulated learning of writing.

## Implications and Conclusion

This study uncovers how a combination of social and cognitive scaffolding sources, including instructor feedback, peer feedback, and revision goals, could shape students’ self-regulated revision. Most revisions (both high and low-level) could be traced from the regulating sources, suggesting the aggregation effects of multiple sources. In terms of the relationship among self-regulated sources, the study found that revision goals were not confined by instructor feedback and peer feedback, and that instructor feedback and peer feedback did not overlap much, which suggests that students can extend beyond environmental regulation from others and approach self-regulation.

The present findings have important pedagogical implications for courses with teaching and learning EFL writing as a primary element. Since these self-regulated sources do contribute to substantial revision, EFL writing instructors may consider using a combination of strategies in multiple draft writing assignment as key sources of information with respect to students’ self-regulated revision intention, in order to facilitate the giving of more effective further direction. Specifically, the goal elicitation strategy used in the current study could be more widely practiced as an internal driving force for further improvement, instead of only using external ones, like instructor feedback and peer feedback. In addition, the current finding adds more empirical evidence to validate how peer feedback could be just as helpful as instructor feedback or even more in carrying out revision. What elements instructor feedback might not cover, for example, grammar errors, peer feedback and revision goals could be a very effective complementary source to help trigger self-regulated revision.

Limitations of the current study need to be addressed in future work. Students’ revision changes have only been traced from three scaffolding sources in one episode during the whole self-regulating process. The longitudinal developmental features of self-regulated learning of writing need to be investigated. For example, how students’ revision goals change across assignments might be a good indicator of their self-regulated learning process. Further, how revision goals, together with instructor and peer feedback work together to scaffold self-regulated learning need experimental studies to validate the effects.

Self-regulated learning of writing acts as the foundational theory for this study, and the study adds empirical evidence to chart the course of the self-regulated process, especially how the goal in combination with external feedback affects self-regulated revision. Further studies could focus on when and how the self-regulation strategies could be used to achieve maximum effects of self-regulation in experimental studies. Other approaches and assistant instruction to eliciting students’ revision goals could be explored to compare and maximize regulation effects. Finally, the causal effects of self-regulated sources on student’s revision and writing also need further investigation.

## Data Availability Statement

The raw data supporting the conclusions of this article will be made available by the authors, without undue reservation.

## Author Contributions

WL and FZ discussed and designed the study. WL collected and analyzed the data, designed figures and tables, and drafted the initial manuscript. WL and FZ revised and proofread the manuscript. Both authors contributed to the article and approved the submitted version.

## Conflict of Interest

The authors declare that the research was conducted in the absence of any commercial or financial relationships that could be construed as a potential conflict of interest.

## Figures and Tables

**APPENDIX A:**
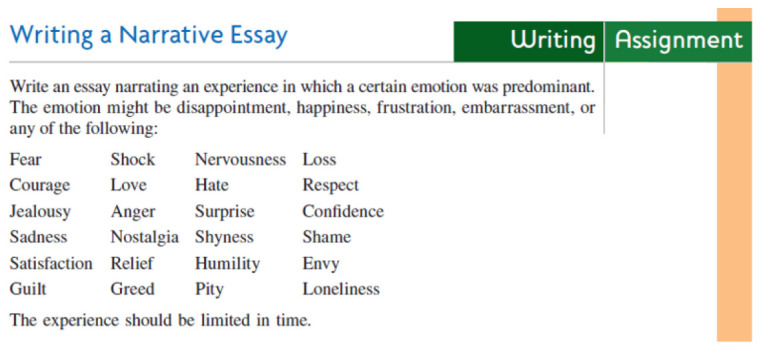
Writing assignment description (adapted from Langan, 2011, p. 223).

## Appendix

**APPENDIX B:** Peer review checklist for narration (adapted from Langan, 2011, p. 225).

**Unity**	
1	Does the writing have a strongly stated topic sentence that clearly identifies a single experience or story that the author is going to recount?
2	Does each sentence help either to keep the action moving or to reveal important things about the characters?
3	Are there sentences or details that do not support the topic sentence and therefore should be eliminated or rewritten?
**Support**	
1	Has the writer included enough vivid, exact details that will help readers experience the event as it actually happened?
2	Has the writer introduced one or more major characters?
3	Does the concluding sentence clearly tie up the story and explain why it is significant?
**Coherence**	
1	Do transitional words and phrases - such as first, later, and then - help make the sequence of events clear?
**Wording/sentence skills**	
1	Has the writer used a consistent point of view throughout the writing?
2	Has the writer used specific rather than general words?
3	Has the writer avoided wordiness and been concise?
4	Are the sentences varied?
5	Has the writer edited for spelling and other sentence skills errors

**APPENDIX C:** Rubrics for rating writing quality.

**Unity**	
7	Very clear central idea, no irrelevant content
6	Between 7 and 5
5	Clear central idea with some irrelevant content
4	Between 5 and 3
3	Vague central idea with much irrelevant content
2	Between 3 and 1
1	Off-topic
**Clarity and support**	
7	The supportive evidence is very specific, typical, and adequate
6	Between 7 and 5
5	The supportive evidence is specific, typical, and adequate
4	Between 5 and 3
3	The supportive evidence is not very specific, typical, or adequate
2	Between 3 and 1
1	Little supportive evidence
**Coherence and cohesion**	
7	The supporting ideas and sentences are well connected, with good transitions and a clear order
6	Between 7 and 5
5	The supporting ideas and sentences connect with each other, though the sentence order is not very clear
4	Between 5 and 3
3	The supporting ideas and sentences are loosely connected, with a few disordered sentences and ideas
2	Between 3 and 1
1	No flow
**Grammar, spelling, wording, and sentence skills**	
7	The low-level dimensions are used very effectively
6	Between 7 and 5
5	The low-level dimensions are used effectively
4	Between 5 and 3
3	Most of the low-level dimensions are used correctly
2	Between 3 and 1
1	The low-level dimensions are used incorrectly
